# A Bayesian model for gene family evolution

**DOI:** 10.1186/1471-2105-12-426

**Published:** 2011-11-01

**Authors:** Liang Liu, Lili Yu, Venugopal Kalavacharla, Zhanji Liu

**Affiliations:** 1 Department of Statistics, University of Georgia, Athens, GA 30602, USA; 2 Department of Biostatistics, Georgia Southern University, Statesboro, GA 30460, USA; 3 Department of Agriculture, Delaware State University, Dover, DE, 19901, USA

## Abstract

**Background:**

A birth and death process is frequently used for modeling the size of a gene family that may vary along the branches of a phylogenetic tree. Under the birth and death model, maximum likelihood methods have been developed to estimate the birth and death rate and the sizes of ancient gene families (numbers of gene copies at the internodes of the phylogenetic tree). This paper aims to provide a Bayesian approach for estimating parameters in the birth and death model.

**Results:**

We develop a Bayesian approach for estimating the birth and death rate and other parameters in the birth and death model. In addition, a Bayesian hypothesis test is developed to identify the gene families that are unlikely under the birth and death process. Simulation results suggest that the Bayesian estimate is more accurate than the maximum likelihood estimate of the birth and death rate. The Bayesian approach was applied to a real dataset of 3517 gene families across genomes of five yeast species. The results indicate that the Bayesian model assuming a constant birth and death rate among branches of the phylogenetic tree cannot adequately explain the observed pattern of the sizes of gene families across species. The yeast dataset was thus analyzed with a Bayesian heterogeneous rate model that allows the birth and death rate to vary among the branches of the tree. The unlikely gene families identified by the Bayesian heterogeneous rate model are different from those given by the maximum likelihood method.

**Conclusions:**

Compared to the maximum likelihood method, the Bayesian approach can produce more accurate estimates of the parameters in the birth and death model. In addition, the Bayesian hypothesis test is able to identify unlikely gene families based on Bayesian posterior p-values. As a powerful statistical technique, the Bayesian approach can effectively extract information from gene family data and thereby provide useful information regarding the evolutionary process of gene families across genomes.

## Background

A gene family is a group of genes with similar sequences and biochemical functions [1-3]. Investigation of the evolution of gene families provides valuable information regarding the evolutionary forces that may have shaped the genomes of species [4-6]. Advancing biotechnology provides a vast amount of data for the studies of gene family evolution. Meanwhile, probabilistic models, describing the evolutionary process of gene families along a phylogenetic tree, significantly facilitate the analyses of gene family data [7-12]. The size of a gene family may expand or contract over time due to gene duplication and loss [8,10,13-15]. The birth and death (BD) model [16-18], which assumes that the size of a gene family follows a birth and death process [8,19-21], is one of the most frequently used models for gene family evolution [7,8,22,23]. Given the phylogenetic tree, the probability distribution of the size of a gene family has been derived under a probabilistic graphical model (PGM) [24]. Parameters in the PGM include the birth and death rate *λ *and the counts of gene copies (i.e., the sizes of ancient gene families) at the internal nodes of the phylogenetic tree. The PGM assumes that the phylogenetic tree is given [5,8,25], though the tree is often estimated from other sources of data. The PGM provides a probabilistic judgment of the hypothesis that different evolutionary forces may have acted on particular gene families or particular lineages of the phylogenetic tree [8]. The PGM can be used to simulate gene family data to evaluate the performance of various computational methods for gene family evolution, including comparative phylogenetic methods [26] that estimate gene duplication and loss events by mapping gene trees onto the species tree [27]. In contrast to comparative phylogenetic methods, the maximum likelihood (ML) method [8] under the PGM is able to estimate the birth and death rate *λ*.

In this study, we develop a Bayesian approach for estimating the birth and death rate λ and the sizes of ancestral gene families at the internal nodes of the phylogenetic tree. Moreover, a Bayesian hypothesis test [28] is developed to identify the gene families that are highly unlikely under the birth and death model. Our major goal is to provide a Bayesian alternative to the ML method for estimating parameters in the birth and death model [8]. Although simulation results suggest that the Bayesian estimates of the model parameters are more accurate than the maximum likelihood estimates, it does not necessarily imply that the Bayesian method developed in this paper, in general, outperforms the ML method. In fact, both methods are useful for making inferences on the evolution of gene families.

## Methods

### A Bayesian model for gene family evolution

Let *X *= {*X_ij_*,*i *= 1,...,*I *and *j *= 1,...,*J*} denote gene family data, where *X_ij _*is the size (the number of gene copies) of gene family *i *for species *j*, *I *is the total number of gene families in the data, and *J *is the number of species. The Bayesian model has the following parameters; *ψ*: the phylogenetic tree; *θ_ik_*: the size of gene family *i *at internal node *k*; and λ: the birth and death rate parameter. We assume that the topology and branch lengths (in millions of years) of the phylogenetic tree are known. The Bayesian model consists of two major components [29]; the prior distribution of model parameters {*λ*,*θ*,*ψ*} and the likelihood function *f*(*X*|*λ,θ,ψ)*, i.e., the probability distribution of gene family data *X *given parameters {*λ*,*θ*,*ψ*}. As the phylogenetic tree is known, the prior distribution of *ψ *is trivial, i.e., the phylogenetic tree with branch lengths is fixed with probability 1. Given the tree *ψ*, we assume that the prior distribution *f*(*λ*|*ψ*) of the birth and death rate λ is uniform (0, 1/max(t)), where max(*t*) is the largest branch length in the tree (see below for the restricted parameter space of λ). We also assume that there is no prior knowledge about *θ *(the counts of gene copies at the internal nodes of the tree), i.e., the prior *f*(*θ*|*λ*,*ψ*) of *θ *is a discrete uniform distribution.

**Figure 1:**
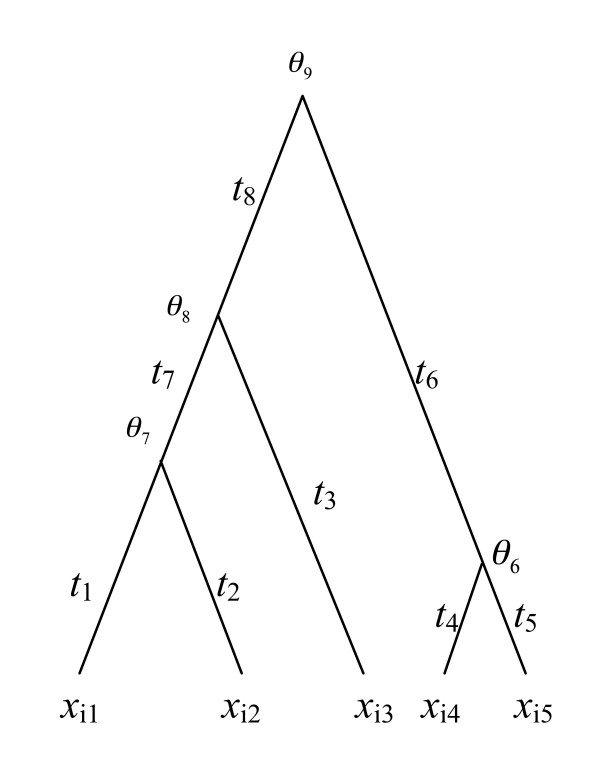
**A birth and death process along the lineages of a phylogenetic tree**. The branch lengths **t**s of the phylogenetic tree are given in millions of years. In the phylogenetic tree, (x**_i_**_1_, x**_i_**_2_, x**_i_**_3_, x*_i_*_4_, x*_i_*_5_) are the sizes of gene family *i *for species 1, 2, 3, 4, and 5, while (*θ_i6_*, *θ_i7_*, *θ_i8_*, *θ_i9_*,) are the sizes of the internal nodes for gene family *i*.

The probability distribution of *X *given parameters {*λ*,*θ*,*ψ*} is derived under the PGM. Let *t_k _*be the length of branch *k *(Figure 1). The counts of gene copies {*x_i_*_1_, *x_i_*_2_, *x_i_*_3_, *x_i_*_4_, *x_i_*_5_} at the tips of the tree represent the sizes of gene family *i *for species 1, 2, 3, 4, and 5, while {*θ_i_*_6, _*θ_i_*_7, _*θ_i_*_8, _*θ_i_*_9,_} are the counts of gene copies at the internal nodes for gene family *i *(Figure 1). Under the BD model, the probability that the number of gene copies changes from *s *(at the parent node *x_p_*) to *c *(at the child node *x_c_*) after time *t *on a particular branch *w *is [8].

(1) $$\begin{array}{c} P_{w}\left\{x_{c}=c|x_{p}=s,t\right\}\\ =\overset{min\left(s,c\right)}{\underset{j=0}{\sum }}\left(\begin{array}{c} s\\ j \end{array}\right)\left(\begin{array}{c} s+c-j-1\\ s-1 \end{array}\right)\alpha^{s+c-2j}{\left(1-2\alpha \right)}^{j} \end{array},$$

where $\alpha =\frac{\lambda t}{1+\lambda t}$ and λ is the birth and death rate parameter. Because (1-2*α*) must be positive, the birth and death parameter λ is subject to a constraint *λ*<1/max(*t*) in which max(*t*) is the largest branch length in the tree. With a complete assignment of all nodes in the tree, the birth and death processes on the different branches are independent of one another. The probability distribution for gene family *i *(denoted by *X_i_*) is thus the product of the probabilities defined in (1) across all branches in the phylogenetic tree, i.e.,

(2) $$f\left(X_{i}|\lambda ,\theta ,\psi \right)=\overset{2J-2}{\underset{w=1}{\prod }}P_{w}\left\{x_{c}=c|x_{p}=s,t\right\},$$

Note that there are (2*J*-2) branches in a *J*-taxon phylogenic tree. Finally, the probability distribution of *X *is equal to the product of the probability densities defined in (2) across all gene families, i.e.,

(3) $$f\left(X|\lambda ,\theta ,\psi \right)=\overset{I}{\underset{i=1}{\prod }}f\left(X_{i}|\lambda ,\theta ,\psi \right).$$

### Bayesian estimation of model parameters

Estimation of the birth and death rate λ and assignments *θ *of the internal nodes is based on the joint posterior probability distribution *f*(*λ*,*θ*|*X*,*ψ*) of λ and *θ*, i.e.,

(4) $$\begin{array}{c} f\left(\lambda ,\theta |X,\psi \right)\\ =\frac{f\left(X|\lambda ,\theta ,\psi \right)f\left(\lambda |\psi \right)f\left(\theta |\lambda ,\psi \right)}{\underset{\left\{\lambda ,\theta \right\}}{\iint }f\left(X|\lambda ,\theta ,\psi \right)f\left(\lambda |\psi \right)f\left(\theta |\lambda ,\psi \right)d\lambda d\theta }. \end{array}$$

As the integral in the denominator of *f*(*λ*,*θ*|*X*,ψ) is analytically intractable, the Metropolis-Hastings algorithm [30,31] is employed to estimate the posterior probability distribution *f*(*λ*,*θ*|*X*,ψ) in (4). The algorithm starts with a set of arbitrary values of parameters λ and *θ*. The value of λ (or *θ*) is then updated at each iteration [32]. The new value *λ*' is accepted with a probability defined by the Hastings ratio *H*,

$$H=min\left\{\frac{f\left(X|\lambda^{\prime },\theta ,\psi \right)f\left(\lambda^{\prime }|\psi \right)f\left(\theta |\lambda ,\psi \right)}{f\left(X|\lambda ,\theta ,\psi \right)f\left(\lambda |\psi \right)f\left(\theta |\lambda ,\psi \right)},\;1\right\}.$$

After the burn-in period, the Metropolis-Hastings algorithm converges to the posterior probability distribution *f*(*λ*,*θ*|*X*,*ψ*). The convergence rate of the Metropolis-Hastings algorithm is largely dependent on the starting values of λ and *θ*. It follows from (1) that given *s *and time *t*, the mean and variance of *c *are equal to (Bailey 1964):

Thus assignment *θ_ik _*(the number of gene copies, or *s *in equation (1)) of internal node *k *for gene family *i *can be consistently estimated by the average count ${\hat{\theta }}_{ik}$ of gene copies at the terminal nodes that are the descendants of node *k*. According to the law of large numbers, ${\hat{\theta }}_{ik}$ is a consistent and unbiased estimator of *θ_ik_*. Additionally, the variance of a transformed random variable $y_{ij}=\frac{X_{ij}}{\sqrt{2{\theta_{i}}^{*}t^{*}}}$, where θ_i_^* ^is the assignment of the tree root for gene family *i *and t^* ^represents the tree height, is equal to *λ_j _*(the average rate along the branches connecting the root and the terminal node of species *j*, because

(6) $$\text{var}\left(y_{ij}\right)=\text{var}\left\{\frac{X_{ij}}{\sqrt{2{\theta_{i}}^{*}t^{*}}}\right\}=\lambda_{j}.$$

The last equality in (6) is derived from (5) by setting *s *= θ_i_*, *t *= t*, and *λ *= *λ_j_*. Equation (6) suggests that *λ_j _*can be consistently estimated by the variance of the transformed data {*y*_1*j*_, *y*_2*j*_,..., *y_Ij _*} for species *j*, i.e.,

(7) $${\hat{\lambda }}_{j}=\frac{1}{I-1}\overset{I}{\underset{i=1}{\sum }}{\left(y_{ij}-{ȳ}_{j}\right)}^{2}.$$

If the assignment of the root for gene family *i *is unknown, θ_i_^* ^in (6) is replaced by its consistent estimate ${{\hat{\theta }}_{i}}^{*}$. When *λ *is constant among all branches of the tree, it is straightforward that the average rate, i.e., $\hat{\lambda }=\frac{1}{J}\overset{J}{\underset{j=1}{\sum }}{\hat{\lambda }}_{j}$is a consistent estimate of *λ*. We use these consistent estimates as the starting values of λ and *θ *to improve the convergence rate of the Metropolis-Hastings algorithm. Convergence of the Metropolis-Hastings algorithm may be assessed by comparing the results from two or more independent runs [33,34]. Running multiple chains, however, will dramatically increase the computational time. More commonly, convergence of the algorithm is evaluated by examining the log likelihood values for a single run [33].

### Posterior Predictive P-value for detecting unlikely gene families

Some gene families may have significantly higher or lower birth and death rates than other families in the dataset. These gene families are highly unlikely to be observed under the BD model that assumes a constant birth and death rate among all gene families. The classical p-value for detecting unlikely gene families depends on the assignment of the tree root [8]. Because the size of a gene family at the root of the tree is unknown in most practical situations, the classical p-value cannot be directly calculated. This is generally called "nuisance parameter problem" (the nuisance parameter is the assignment of the root) [28,35]. To overcome this problem, Hahn et al. [4] proposed to compute the maximum conditional p-value among all possible assignments of the root. Although Hahn et al. [4] have demonstrated that the maximum conditional p-value can be used to detect unlikely gene families, it should be noted that the maximum conditional p-value is no longer the tail-area probability as intended in classical approaches [28].

Posterior Predictive P-value (PPP) is the Bayesian alternative to the classical p-value [28]. The Bayesian P-value can be used to evaluate statistical significance for the (alternative) hypothesis that the observed size of a particular gene family is highly unlikely under the BD model. Here the null hypothesis is that the BD model can explain the observed size of the gene family across species. The Bayesian P-value is defined as the average p-value $p_{\lambda ,\theta ,H_{0}}$ over the posterior distribution *f*(*λ*,*θ|X,H*_0_) under the null hypothesis (*H*_0 _), i.e.,

(8) $$PPP=\underset{\Omega }{\int }p_{\lambda ,\theta ,H_{0}}\times f\left(\lambda ,\theta |X,H_{0}\right)\;d\lambda d\theta .$$

In (8), Ω represents the space of parameters *λ *and *θ*. The conditional p-value $p_{\lambda ,\theta ,H_{0}}$ is the probability that the likelihood score *f*(X_i_*|*λ*,*θ*,*ψ*) of a random gene family X_i_^* ^is less than that of the observed family *X_i_*, i.e.,

$$p_{\lambda ,\theta ,H_{0}}=\mathrm{Pr}\left\{f\left({X_{i}}^{*}|\lambda ,\theta ,\psi \right)<f\left(X_{i}|\lambda ,\theta ,\psi \right)\right\}.$$

A random gene family X_i_^* ^is generated from the BD model at each cycle of the MCMC algorithm. The PPP of gene family *X_i _*is estimated by the proportion of cycles at which the likelihood score *f*(X_i_*|*λ*,*θ*,*ψ*) is less than *f*(*X_i_*|*λ*,*θ*,*ψ*) [28]. Under the null hypothesis, PPP is expected to be near 0.5 [28]. Extreme PPPs (close to 0 or 1) imply that gene family *X_i _*is highly unlikely to be observed under the BD model. Moreover, a gene family with a slow birth and death rate tends to have a higher likelihood score than a gene family with a fast rate. Thus a small PPP (close to 0) indicates that the birth and death rate of the gene family is significantly greater than those of other gene families. A large PPP (close to 1) implies that the birth and death rate of the gene family is significantly less than the rates of other gene families.

### Testing homogeneous birth and death rates among branches of the tree

The hypothesis of homogeneous birth and death rates among branches of the tree can be tested under the maximum likelihood framework [1,27,36]. Under the Bayesian framework, the evidence for supporting the null hypothesis (H_0_) against the alternative hypothesis (H_1_) is evaluated by the Bayes Factor [37], $BF=\frac{f\left(X|H_{1}\right)}{f\left(X|H_{0}\right)}$, where *f*(*X*|*H*_0_) is the marginal likelihood under the null hypothesis (homogeneous rates) and *f*(*X*|*H*_1_) is the marginal likelihood under the alternative hypothesis (heterogeneous rates). In general, *Ln*(*BF*)>10 [38] is interpreted as strong evidence for supporting the alternative hypothesis (heterogeneous rates).

## Results

### Simulation

**Figure 2:**
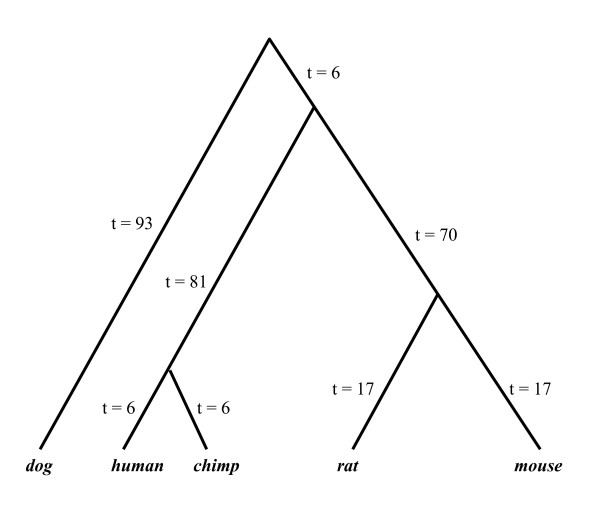
**The phylogenetic tree used in the simulation study**.

Gene family data were simulated from the PGM with a phylogenetic tree of six primates (Figure 2) specified in an example file in CAFE [39]. We assumed a constant birth and death rate among all branches in the phylogenetic tree. Three simulations were conducted with λ = 0.001, 0.005, 0.01 respectively. The simulated datasets were analyzed by the Bayesian and ML methods to estimate λ and the proportions of gene families that showed expansion, contraction, and no change along the eight branches of the phylogenetic tree. The ML analysis was conducted in CAFE [39]. The simulations were repeated 100 times. For the Bayesian analysis, the MCMC algorithm ran for 1000000 iterations. The log-likelihood score approached stationarity at the 100000^th ^iteration for all the 10 simulations randomly chosen for convergence diagnosis. We discarded the initial 200000 iterations as burn-in. To evaluate the performance of the two methods, we calculated the estimation errors of the Bayesian and ML estimates ($\hat{\lambda }$ ) of λ. The estimation error of $\hat{\lambda }$ is equal to $\sqrt{\frac{1}{w}\overset{w}{\underset{i=1}{\sum }}{\left({\hat{\lambda }}_{i}-\lambda \right)}^{2}}$, where *w *is the number of simulations and ${\hat{\lambda }}_{i}$ is the estimate of λ for the *i*^th ^simulation. Similarly, we calculated the error of estimating the proportions of gene families that showed expansion, contraction, and no change on the eight branches of the tree.

**Figure 3:**
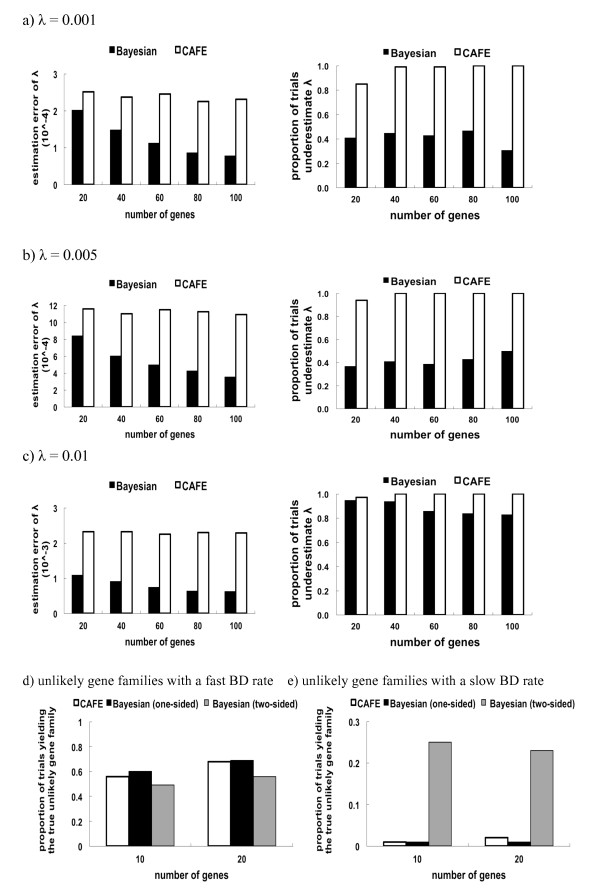
**Simulation results**. The estimation errors of the Bayesian and ML estimates of λ are calculated for the simulations with a) λ = 0.001, b) λ = 0.005, and c) λ = 0.01. The proportion of trials yielding the true unlikely gene family is reported when the unlikely gene family is simulated with d) a fast birth and death rate or with e) a slow birth and death rate.

The simulation results show that the estimation error of the Bayesian estimate of *λ *is less than that of the maximum likelihood estimate (MLE) for all three simulations with *λ *= 0.001, *λ *= 0.005, and *λ *= 0.01 (Figure 3a-c). It suggests that the Bayesian method outperforms the maximum likelihood method in estimating the birth and death rate *λ*. The ML method appears to consistently underestimate *λ *because the proportion of trials underestimating *λ *approaches 1.0 when the number of gene families increases (Figure 3a-c). In contrast, the Bayesian method produces a more unbiased estimate of *λ *when the true value of *λ *is relatively small (*λ *= 0.001, 0.005) (Figure 3a-b). For a large *λ *(*λ *= 0.01), the Bayesian method tends to underestimate the value of *λ*, but the proportion of trials underestimating *λ *appears to decrease as the number of gene families increases (Figure 3c). The simulation results also suggest that the Bayesian method outperforms the ML method in estimating the proportion of gene families that showed expansion, no change, or contraction on the eight branches of the phylogenetic tree (Table 1).

**Table 1:** The estimation error of the proportions of gene families that showed expansions, contractions, and no change.

	λ = 0.001		λ = 0.005		λ = 0.01	
# of gene families	Bayesian	CAFE	Bayesian	CAFE	Bayesian	CAFE
20	0.07	0.138	0.088	0.184	0.089	0.214
40	0.048	0.105	0.062	0.148	0.063	0.179
60	0.032	0.089	0.051	0.134	0.052	0.170
80	0.032	0.084	0.045	0.130	0.045	0.170
100	0.03	0.081	0.039	0.126	0.032	0.164

Gene family data were simulated from the birth and death model with λ = 0.001, 0.005, 0.01 respectively. The Bayesian model and CAFE were then applied to the simulated data to estimate the proportions of gene families that showed expansions, contractions, or no change. The estimation error is equal to the square root of the mean squared error of the estimated proportions of expansions, contractions, and no change. In general, the estimation error decreases as the number of gene families increases.

Additional simulations were carried out to compare the performance of the hypothesis tests based on the Bayesian p-value and the maximum conditional p-value. A total of 9 gene families were simulated using the phylogenetic tree in Figure 2 with *λ *= 0.001. Another gene family was generated from the same phylogenetic tree with a higher birth and death rate *λ *= 0.005 and treated as the unlikely gene family. This represents the scenario that the unlikely gene family has a faster birth and death rate than other gene families. We also considered the scenario where the unlikely gene family has a slower birth and death rate than other gene families. The unlikely gene family was generated with a birth and death rate *λ *= 0.001, while other gene families were generated with *λ =*0.005. The simulated gene families were analyzed by the Bayesian and ML methods (the ML method was implemented in CAFE) respectively to identify unlikely gene families. We carried out two Bayesian hypothesis tests. The one-sided Bayesian hypothesis test identified an unlikely gene family if PPP < 0.1, while the two-sided Bayesian hypothesis test identified an unlikely gene family if PPP < 0.1 or PPP > 0.9. Because a small PPP is associated with the unlikely gene families that have a fast birth and death rate, we expect that the one-sided Bayesian test (PPP < 0.1) is able to identify unlikely gene families with a high birth and death rate (the first scenario described above). However, the one-sided Bayesian test is incapable of identifying unlikely gene families with a slow birth and death rate (the second scenario). In contrast, the two-sided Bayesian hypothesis test works for both scenarios. The type I error was set 0.05 for both Bayesian and classical hypothesis tests. The simulations were repeated 100 times and we calculated the proportion of trials yielding the true unlikely gene families. Finally, we increased the number of simulated gene families from 10 to 20 (including one unlikely gene family) to investigate the effect of the sample size (the number of gene families) on the performance of the Bayesian and classical hypothesis tests.

Overall, the hypothesis tests based on the Bayesian (one-sided and two-sided) and maximum conditional p-values perform almost equally well in identifying the unlikely gene families with a fast birth and death rate (Figure 3d). However, CAFE and the one-sided Bayesian hypothesis test perform poorly in detecting unlikely gene families with a slow birth and death rate (Figure 3e). In contrast, the two-sided Bayesian hypothesis test, as we expected, is capable of identifying gene families with a slow birth and death rate, though the discovery rate is rather low (Figure 3e).

### Real data analysis

The Bayesian model was applied to a gene family dataset generated from five *Saccharomyces *(*S. bayanus*, *S. kudriavzevii*, *S.mikatae*, *S.paradoxus*, *S.cerevisiae*) genomes. The dataset contains 3517 gene families. The phylogenetic tree was given by Hahn et al. [8]. The MCMC algorithm ran for 10,000,000 generations. The log-likelihood score reached stationarity at the 5,000,000^th ^generation. With the assumption of a constant birth and death rate along the lineages of the phylogenetic tree, the Bayesian analysis for the yeast dataset estimated the birth and rate $\hat{\lambda }=0.00213$, which is close to the maximum likelihood estimate $\hat{\lambda }=0.0023$ in the previous study [8]. However, the consistent unbiased estimates (defined in equation (7)) of the birth and death rates along the lineages leading to the five extant species are 0.004, 0.0046, 0.0028, 0.0025, 0.0038 respectively, indicating that the homogeneous rate model may not be able to adequately explain the yeast dataset. The Bayesian analysis of model selection described in the previous selection confirmed that the BF ( > 100) strongly favors the heterogeneous rate model. Thus the analysis of the yeast dataset is based on the Bayesian heterogeneous rate model.

Unlikely gene families were identified on the basis of their PPP values under the Bayesian heterogeneous rate model. A gene family is identified as an unlikely family if PPP < 0.01 or PPP > 0.99 (the corresponding type I error is < 0.005). A large PPP (> 0.99) suggests that the birth and death rates of the unlikely gene families on some branches of the phylogenetic tree are significantly smaller than those of other gene families. A small PPP (< 0.01) suggests that the birth and death rates of unlikely gene families on some branches are significantly larger than those for other gene families. The two-sided Bayesian hypothesis test suggests that 2263 gene families have PPP values > 0.99. It is not a surprise because all these gene families have no change in size across five yeast species, extremely unlikely to be observed under the BD model. This result suggests that the yeast dataset may reflect two different evolutionary patterns. A majority of gene families (2263) have no change in size across five *Saccharomyces *species, suggesting a very slow birth and death rate (close to 0), while the sizes of the remaining 1254 gene families are distinct across species, suggesting a relatively fast birth and death rate. It would be more appropriate to analyze the two groups of gene families separately. It is, however, unnecessary to analyze the 2263 gene families with no change in size because these gene families obviously support a very slow birth and death rate *λ*.

**Figure 4:**
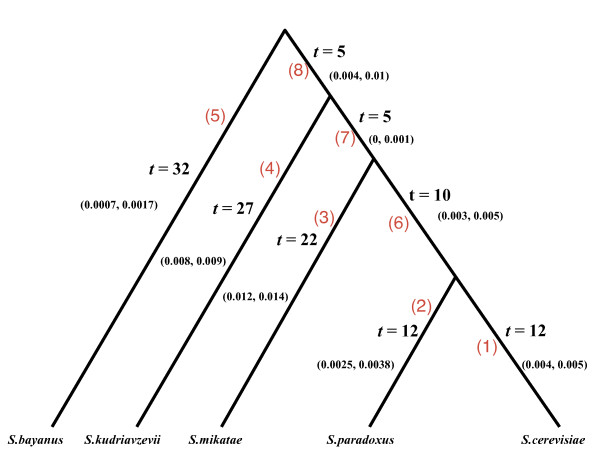
**The estimates of the birth and death rates on the branches of the phylogenetic tree for 1254 gene families of the five yeast species**. The birth and death rates were estimated under the Bayesian heterogeneous rate model. The interval on each branch is the 95% credible interval for the birth and death rate λ. The branch lengths **t **in the tree are given in millions of years [4]. The branch numbers are highlighted in red.

We analyzed the remaining 1254 gene families under the Bayesian heterogeneous rate model. The 95% Bayesian credible intervals for the birth and death rates on the eight branches of the phylogenetic tree suggest that the rates on the branches leading to the species *S.mikatae *and *S. kudrizvzevii *are significantly higher than the rates on other branches (Figure 4). Moreover, there is a clear pattern of expansion on the two branches leading to *S. kudrizvzevii *and *S. mikatae *(Table 2), which agrees with the previous result [8] except that the total number of gene families in the current study is 1254 while it was 3517 in the previous study. The expansion pattern on the branches leading to *S. mikatae *and *S. kudrizvzevii *is consistent with the fast rates estimated for these branches (Figure 4). The Bayesian analysis under the heterogeneous rate model identified 11 unlikely gene families (PPP < 0.05) (Table 3), in contrast to 58 unlikely gene families found in the previous study [8]. Only 4 of the most significantly unlikely gene families (Table 2 in Hahn et al. [8]) found in the previous study are confirmed by the Bayesian analysis. The Dihydrourdine and alpha/beta hydrolase families, (2(2(**6**(2 2)))) and (1(1(**6**(1 1)))), were identified as unlikely gene families in the previous study, because the numbers of gene copies of species *S. mikatae *(highlighted in the Newick notation) for these gene families are significantly greater than those of other species. The Bayesian analysis for 1254 gene families under the homogeneous rate model identified alpha/beta hydrolase (1(1(**6**(1 1)))) as an unlikely gene family, but not Dihydrourdine. It indicates that the difference is probably due to the exclusion of 2263 gene families in the Bayesian analysis. Interestingly, neither Dihydrourdine nor alpha/beta hydrolase were identified as unlikely families by the Bayesian analysis under the heterogeneous rate model. In contrast to the homogeneous rate model, the heterogeneous rate model estimates a relatively high birth and death rate (Figure 4) on the branch leading to species *S. mikatae*, which can explain the observed large number of gene copies for species *S. mikatae*. Thus alpha/beta hydrolase is not identified as an unlikely gene family under the heterogeneous rate model.

**Table 2:** The Bayesian estimates of the numbers of gene families in the reduced yeast dataset (1257 gene families) that showed expansions, no change, or contractions on the eight branches of the phylogenetic tree in Fig.4.

Branch number	Expansions	No change	Contractions
1 (t = 12)	84	1120	50

2 (t = 12)	48	1129	77

3 (t = 22)	616	510	128

4 (t = 27)	496	635	123

5 (t = 32)	51	1107	96

6 (t = 10)	36	1126	92

7 (t = 5)	3	1146	5

8 (t = 5)	50	1134	70

Numbers in the first column are the branch numbers highlighted in Fig. 4.

**Table 3:** The most unlikely gene families identified by the Bayesian hypothesis test.

Family ID	Gene family	PPP
3	(2 (8 (15 (34 83))))	0.000

18	(17 (14 (15 (1 5))))	0.000

28	(1 (3 (3 (2 34))))	0.000

13	(7 (16 (7 (20 17))))	0.002

34	(5 (11 (14 (4 2))))	0.003

6	(15 (33 (24 (30 31))))	0.004

397	(1 (1 (2 (1 5))))	0.006

77	(2 (5 (4 (7 4))))	0.019

256	(1 (2 (7 (1 1))))	0.019

89	(2 (9 (4 (2 2))))	0.021

262	(1 (4 (4 (1 1))))	0.025

## Discussion

Simulation results suggest that the maximum likelihood method tends to underestimate the birth and death rate, while the Bayesian approach is able to produce more accurate estimates of the birth and death rate and other parameters in the BD model. It is not intended in this paper, however, to claim that the Bayesian method is, in general, superior to the maximum likelihood method in estimating model parameters. There might be some cases for which the maximum likelihood method outperforms the Bayesian method and provides more accurate estimates of parameters in the BD model. It demands an extensive simulation study and a sufficient number of empirical data analyses to get a clear picture of how the two methods perform in various situations, which is certainly beyond the scope of this paper.

Recently, Cohen and Pupko [18] developed several probabilistic-evolutionary models for analyzing gene family data. These models assume that the evolution of gene family content follows a continuous time two-state Markov process. The models coupled with stochastic mapping are able to identify horizontal gene transfer events on the lineages of the phylogenetic tree [18]. These models allow the gain and loss rates to vary across gene families [18,40]. Similarly, the Bayesian model developed in this paper can be extended to handling variable rates over gene families by assuming a probability distribution for the gene-family-specific rates.

Choosing the appropriate prior distribution for model parameters is always challenging in Bayesian analyses. A non-informative prior is desirable if there is no prior knowledge about the probability distribution of parameters, but it is often difficult to find a non-informative prior for model parameters. It is reasonable to specify a flat prior (uniform distribution, see the section "A Bayesian model for gene family evolution") for parameters *λ *and *θ *if there is no prior information available for *λ *and *θ*. Alternatively, an informative prior may be used in the Bayesian analysis of gene family data. Nevertheless, concerns about the choice of prior distribution will be greatly alleviated when gene family data, especially those from genomic studies, have a large sample size (for example, the yeast dataset analyzed in this paper involves 3517 gene families).

Both ML and Bayesian methods involve intensive computation. It is unfair, however, to directly compare the computational time for the ML and Bayesian methods because the ML method (implemented in CAFE) produces only the point estimates of model parameters, while the Bayesian method estimates the posterior probability distribution of model parameters. Thus we here only provide the computational time for the Bayesian method (Table 4). The computational time for running the Bayesian analysis for 10000 iterations (conducted on a Lenovo notebook T61) increases linearly with respect to the number of gene families and the number of species. However, the MCMC algorithm will probably need much more than 10000 iterations in order to converge when there is a large number of species in the dataset. For example, the Bayesian analysis for the yeast dataset took about 24 hours (for 10000000 iterations) on a Mac computer (2.16 GHz Intel Core 2 Duo, 1 GB of RAM).

**Table 4:** The computational time (seconds) for running the Bayesian analysis (10000 iterations) on a Lenovo notebook T61 (Intel 2 Duo CPU, 2.4 GHz, 2.48 GB of RAM).

number of gene families	5 species	10 species	20 species
10	11	22	42

20	20	38	52

40	40	64	104

The Bayesian p-value appears to be useful in identifying unlikely gene families. It should be noted, however, that neither the classical p-value nor the Bayesian p-value represents the probability that the null hypothesis is true. Thus they do not provide direct evidence for accepting or rejecting the null hypothesis. The Bayesian p-value can be interpreted as a measure of discrepancy between the observed data and those expected from the assumed probabilistic model under the null hypothesis. Gene families with small (typically < 0.05) or large (> 0.95) Bayesian p-values can be regarded as outliers (or unlikely gene families), which are unlikely to be observed under the null hypothesis. The Bayesian p-value provides a general way to handle the problem of nuisance parameters [28]. Regardless of the type of p-values (the Bayesian p-value or the maximum conditional p-value) in use, the hypothesis test for unlikely gene families does not appear to have much power when the unlikely gene family has a slow birth and death rate (Figure 3e).

## Conclusions

Accurately estimating the birth and death rate as well as the numbers of gene copies at the internal nodes of the phylogenetic tree is the major goal of the statistical analyses of gene family data. In this paper, we develop a Bayesian approach for estimating these parameters from gene family data. The results of the simulation study and the empirical data analysis suggest that the Bayesian method can accurately estimate the parameters in the BD model. The source code for implementing the Bayesian analysis is written in C and available at http://code.google.com/p/begfe.

## Authors' contributions

LL and LY developed the method and conducted the analyses. LL, LY, VK and ZL drafted the manuscript. All authors read and approve the final manuscript.
